# The PANEN nomogram: clinical decision support for patients with metastatic pancreatic neuroendocrine neoplasm referred for peptide receptor radionuclide therapy

**DOI:** 10.3389/fendo.2025.1514792

**Published:** 2025-06-24

**Authors:** Aviral Singh, Sebastian Sanduleanu, Harshad R. Kulkarni, Thomas Langbein, Philippe Lambin, Richard P. Baum

**Affiliations:** ^1^ Theranostics (Oncology), GenesisCare Pty Ltd, Murdoch, WA, Australia; ^2^ Department of Precision Medicine, GROW—School for Oncology and Developmental Biology, Maastricht University, Maastricht, Netherlands; ^3^ Department of Nuclear Medicine, Fiona Stanley Hospital, South Metropolitan Health Service, Murdoch, WA, Australia; ^4^ Department of Radiology and Neuroradiology, GFO Clinics Troisdorf, Academic Hospital of the Friedrich-Wilhelms-University Bonn, Troisdorf, Germany; ^5^ Bold Advanced Medical Future (BAMF) Health, Grand Rapids, MI, United States; ^6^ Clinic for Nuclear Medicine, Zentralklinik Bad Berka, Bad Berka, Germany; ^7^ Advanced Theranostics Center for Radiomolecular Precision Oncology, CURANOSTICUM Wiesbaden-Frankfurt, HELIOS DKD Klinik, Wiesbaden, Germany

**Keywords:** pancreatic neuroendocrine neoplasm, clinical decision support nomogram, peptide receptor radionuclide therapy, pedictict overall survival, machine learning

## Abstract

**Introduction:**

Patients with pancreatic neuroendocrine neoplasms (P-NEN) may benefit from peptide receptor radionuclide therapy (PRRT). Prediction of overall survival (OS) using statistical models has the potential to guide treatment decisions. In this study, we have generated a clinicopathological and imaging parameter-based internally validated nomogram of patients who received PRRT for metastatic P-NEN to facilitate treatment decision support for the clinical management of such patients.

**Patients and methods:**

We reviewed 447 pancreatic NEN patients treated with PRRT. Clinical variables for the prediction of overall survival (OS) included age, gender, Karnofsky performance score (KPS), weight loss, hepatomegaly, time from diagnosis to first PRRT (days), tumor functionality, presence of Hedinger syndrome, presence of liver metastases, presence of bone metastases, presence of lung metastases, alkaline phosphatase, 2-deoxy-2-[18F]fluoro-D-glucose ([_18_F]FDG) positron emission tomography (PET) scan positivity, erythrocytes, platelets, creatinine clearance, leucocytes, and histologic grade of tumor differentiation based on KI-67 staining. A random survival forests (RSF) method was used to construct a model with an optimal number of clinical variables. The model was developed on 80% of the data and tested on the remaining 20% of the data. Performance of prediction was calculated using the c-index, a generalization of the area under the ROC curve (AUC) for survival models.

**Results:**

Median follow up time was 2045 days (min 136 days, max 10329 days). Time from diagnosis to 1^st^ PRRT, alkaline phosphatase, KPS, hepatomegaly, weight loss, [_18_F]FDG-PET scan positivity, Ki-67% derived histologic grade, lung metastases, age, presence of bone metastases, platelet count, erythrocyte count, creatinine clearance, hemoglobin, presence of functioning tumor, creatinine, and gender, were in order of importance, all independent predictors for overall survival. The development set c-index was 0.86, while the test set c-index was 0.82. A nomogram was constructed based on the optimal number of clinical parameters selected in the RSF model.

**Conclusion:**

This study proposes an internally validated nomogram (PANEN-N) to accurately predict overall survival for P-NEN patients following PRRT, which could be used for patient counseling to facilitate informed and shared decision support in daily clinical practice as well as for generating new hypotheses.

## Introduction

Pancreatic neuroendocrine neoplasms (P-NEN), previously also known as islet cell tumors, are a rare group of neoplasms that account for less than 3% of all pancreatic tumors (1).

The majority of P-NENs (70-90%) are non-functioning (i.e., not associated with a hormonal syndrome such as in the case of insulinomas, glucagonomas, gastrinomas, somatostatinomas, and VIPomas) (2), posing a challenge in the diagnosis of these tumors at an early stage.

While the majority of these neoplasms are sporadic, they may be associated with a number of genetic syndromes such as multiple endocrine neoplasia-1 and von Hippel-Lindau syndrome (3).

Based on the 2017 World Health Organization (WHO) classification, P-NEN are divided into well-differentiated P-NETs: grade 1 (G1), Ki-67 <3% and/or mitotic rate <2 mitoses/2 mm2; grade 2 (G2), Ki-67: 3–20% and/or mitotic rate 2–20 mitoses/2 mm2; grade 3 (G3), Ki-67 > 20%; and poorly-differentiated pancreatic neuroendocrine carcinoma (P-NEC) including small-cell type (SCNEC) and large-cell type (LCNEC), Ki-67 > 20% and/or mitotic rate >20/2 mm2 (4).

The incidence rates of P-NEN have been increasing worldwide, which is most likely caused by the increased detection of asymptomatic disease on cross-sectional imaging and endoscopy performed for other indications (5).

Novel biomarkers, such as circulating DNA, genomic and transcriptomic profiles, mRNA and circulating tumor cells, are being developed; however, still only available in the pre routine clinical setting (6, 7). Blood sampling or liquid biopsy for the assessment of neuroendocrine gene transcripts have demonstrated significant diagnostic and prognostic potential in recent studies, such as the NET-test, nevertheless these currently are not available in all countries for regular clinical application (8, 9).

The introduction of various modern imaging modalities has improved tumor localization as well as staging and restaging of neuroendocrine neoplasms. Although, Ga-68 labeled somatostatin receptor (SSTR) PET/CT has been widely used in Europe for the past two decades, the FDA only approved the use of PET/CT imaging with Ga-68 labeled DOTATATE in June 2016 (10). Gallium-68 (^68^Ga)-edotreotide has been authorized for molecular imaging of gastroenteropancreatic neuroendocrine (GEP-NET) tumors in the European Union since December 2016 (11).

A ‘NETPET’ grade has been proposed as a promising prognostic imaging biomarker in NEN with PET scans using [_18_F]FDGand SSTR imaging agents, which permits assessment of the glycolytic as well as somatostatin receptor status of the tumor using this dual radiotracer imaging in each patient to describe tumor heterogeneity, and thereby highlighting the more aggressive phenotype of NEN in that specific patient (12).

Historically, the management of P-NEN has been a complicated task mainly due to the heterogeneity of these tumors. The mainstay of treatment has been surgical excision of small and localized tumors. However, the majority of patients recur, even if the local resection is complete (13). Additional systemic treatments include biotherapy with somatostatin analogues, mTOR inhibitors (everolimus), multi-tyrosine kinase inhibitor (e.g., sunitinib), systemic chemotherapeutic agents such as capecitabine and temozolomide, liver metastases directed therapies e.g., chemoembolization, and receptor mediated radionuclide treatment strategies (14). Peptide receptor radionuclide therapy (PRRT) has been practiced for over two decades on a compassionate use basis in Europe as well as certain other countries (15, 16). However, the first phase-III, prospective, randomized controlled trial (NETTER-1) comparing [_177_Lu]Lu_3+_ labeled SSTR analog radionuclide therapy with high-dose cold SSTR-analog in gastroenteropancreatic neuroendocrine tumors (GEP-NET), demonstrated a significantly higher progression-free survival (PFS) in the [_177_Lu]Lu-DOTATATE group with minimal adverse effects and excellent tolerability (17), which subsequently led to the approval of PRRT in GEP-NETs by the Food and Drug Administration (FDA) (18) and the European Medicines Agency (EMA) (19). The recently published NETTER-2 trial (20) demonstrated that treatment with PRRT using [_177_Lu]Lu-177 DOTA-TATE in the first line setting versus standard of care (control arm) significantly improved median progression-free survival (PFS) and demonstrated clinically meaningful objective response rates (ORR) in patients with higher grades (grade 2 and 3) GEP-NET. In the study, 54.4% patients had P-NEN and 29.2% patients had small-bowel NEN. Median PFS (22.8 months vs 8.5 months) and ORR (43.0% vs 9.3%) were significantly higher in patients in the treatment arm when compared to patients in the control arm, respectively. This reaffirms the therapeutic efficacy of PRRT in P-NEN even at the initial stages of therapy.

Over the past years, studies have successfully demonstrated the clinical applicability of a mathematically validated nomogram, may provide objective assessment for the surgical management of P-NEN patients (21), as well as for small intestine neuroendocrine tumor patients being considered for either surgical management or somatostatin analogue therapy (22).

With regards to the application of PRRT, there remains a lack of a similar structured and validated clinical and patient decision support system, which can be applied more universally and used widely in everyday clinical practice.

In this study, we have generated a clinicopathological as well as imaging parameter-based internally validated nomogram of patients who received PRRT for metastatic P-NENs in order to facilitate treatment decision support for the clinical management in this group of patients. This nomogram, called the PANEN Nomogram (PANEN-N), is based on the analysis of the currently largest number of P-NEN patients treated with PRRT at a single center.

## Materials and methods

### Patient cohort

In this single center retrospective cohort study from November 2002 to September 2019, a total of 447 patients with metastatic G1 to G3 P-NEN (M 250 (56%), F 197 (44%); age range 19–88 years, mean age 62 years), who underwent PRRT at Zentralklinik Bad Berka, Germany, were retrospectively reviewed.

Patient selection for PRRT was in accordance with the published guidelines for PRRT (23), including relevant clinical parameters such as life expectancy of more than 6 months, somatostatin receptor positive pancreatic NEN, and adequate renal function and bone marrow reserve. The diagnosis of P-NEN was confirmed based on histopathological reports performed on the tumor tissue of the respective patients. The final decision to perform PRRT was made by the multidisciplinary neuroendocrine tumor board established and regularly audited by the European Neuroendocrine Tumor Society (ENETS). The baseline demographics and clinical characteristics of the patients are listed in **Table 1**.

**Table 1:** Baseline demographics and clinical characteristics of the patients.

Variable name	Count	Percentage
Age (years (± SD))	62 (± 12)	
Gender		
Male (n)	250	56%
Female (n)	197	44%
Tumor grade (based on Ki67 proliferation index)		
G1	75	17%
G2	208	47%
G3	46	10%
Unavailable	118	26%
Tumor functional status		
Functioning tumors (n)	98	21.9%
Non-functioning tumors (n)	349	78.1%
Previous surgery		
Excision of liver metastases	262	59%
Pancreatectomy	163	36%
Small intestine resection	7	2%
Large intestine resection	15	3%
None	118	26%
Other (not tumor-specific)	121	27%
Previous systemic treatment		
Chemotherapy	131	29%
Everolimus	3	1%
Interferon	24	5%
Lanreotide/Somatuline	8	2%
Sandostatin	163	36%
Other	3	1%
None	115	26%
Karnofsky performance score		
<= 50	24	5%
60	18	4%
70	26	6%
80	79	18%
90	207	46%
100	92	21%
Unavailable	1	0%
Median survival (days, (± SD))	1011 (± 1002)	

**Figure 1:**
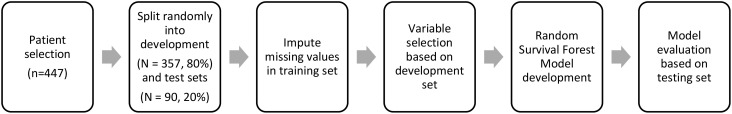
Schematic overview of the Random Survival Forest (RSF) model development process used in this study.

In total 447 patients received PRRT and were included in the final analysis. Multivariate analyses for overall survival were based on Random Survival Forests (RSF), an ensemble tree method for analysis of right-censored survival data. The model was learned on a randomly selected 80% of the data and tested on the remaining 20% of data. Model results were expressed by the c-index. A schematic overview of the model development process used in this study is shown in **Figure 1**.

### Statistical analysis

Statistical analyses were conducted in R (version 3.4.0). A two-sided p-value cut-off of 0.05 was set to determine statistical significance. The prognostic value of the individual clinical features was evaluated using concordance index (CI) with the survival package (Therneau T (2015)). A Package for Survival Analysis in R. version 2.38, URL: https://CRAN.R-project.org/package=survival) and randomForestSRC package (Ishwaran H (2017) Fast Unified Random Forests for Survival, Regression and Classification (RF-SRC) version 2.9.1, URL: https://cran.r-project.org/web/packages/randomForestSR). Nomograms were constructed with the ‘nomogram’ function in the ‘rms’ package and the ‘DynNom’ package which generates a dynamic nomogram application for a variety of statistical models to allow a reader to interact with the model in a user-friendly manner as a standalone application or web-based interface.

Multivariable clinical Random Survival Forest (RSF) models were generated based on selecting all clinical features with a relative feature importance >0. Variable importance was computed based on the decrease of node impurity when the covariate in question is considered for the splitting.

Random Survival Forest (RSF) strictly adheres to the prescription laid out by Breiman (2003) and requires considering the outcome (splitting criterion used in growing a tree must explicitly involve survival time and censoring information) in growing a random forest model. Further, the predicted value for a terminal node in a tree, the resulting ensemble predicted value from the forest, and the measure of prediction accuracy must all properly incorporate survival information.

After selecting the important variables in the RSF analysis, the nomogram was based on a Cox proportional hazards model with the selected variables. The reason for this is that unlike traditional parametric models (such as the Cox proportional hazards model), RSF does not provide explicit coefficients for each predictor variable. Instead, RSF generates survival trees and makes predictions based on an ensemble of these trees which makes it challenging to create a simple, interpretable nomogram that directly translates the RSF’s predictions into probabilities.

### Variable selection

Variables included in the analysis were age, gender, KPS, weight loss, hepatomegaly, time from diagnosis to first PRRT (days), tumor functionality, presence of Hedinger syndrome, presence of liver metastases, presence of bone metastases, presence of lung metastases, alkaline phosphatase, [_18_F]FDG scan positivity, erythrocytes, platelets, creatinine clearance, leucocytes, and histologic grade of tumor differentiation based on KI-67 staining. These parameters were established at the time of decision to commence PRRT for each patient.

## Results

In total n=250 male (56%) and n=197 female participants (44%) were included in this retrospective cohort analysis. Median survival time for the entire cohort was 33.2 ± 32.9 months.

Biopsy-based tumor grades were available for n=329 (74%) patients and varied between G1 (17%), G2 (47%), and G3 (17%) according to Ki-67 proliferation index. Out of the 447 patients, 98 (21.9%) had functioning tumors and remaining 349 (78.1%) had non-functioning tumors. Within this cohort, (47%) 208 patients had prior surgical intervention related to their disease, out of which more than one-third (36%) patients had undergone pancreatectomy. With regards to other treatments, n=171 (38%) patients had received prior treatment with long-acting somatostatin analogues, particularly, Octreotide (Sandostatin^®^ LAR^®^) and Lanreotide (Somatuline^®^ AG^®^) in n=163 (36%) and n=8 (2%) patients, respectively. Previous chemotherapy was administered in n=131 (31%) patients. Interestingly, the mTOR inhibitor, Everolimus had been the therapy of choice for a meager 1% patients, which could probably relate to the approval of Everolimus for the treatment of unresectable or metastatic, well- or moderately-differentiated neuroendocrine tumors (NET) of pancreatic origin in adults with progressive disease following the results of the RADIANT-3 trial from 2011 (24) and probably also due to the comparatively more severe toxicity profile. Other therapeutic options such as interferon-alpha were used in lesser number of patients. The baseline demographics and clinical characteristics of the patients are reported in **Table 1**.

Median follow up time was 2045 days (min 136 days, max 10329 days). In total 308 patients died by the end of follow up. Three-hundred and fifty-seven randomly selected patients (80%) were included in the development set, while 90 patients were held out for the test set (20%).

In total n=286 patients had undergone an [_18_F]-FDG-PET study.

In total 17 variables were selected based on basis of their relevant importance in the RSF analysis. The development set c-index was 0.86, while the test set c-index was 0.82.

**Figure 2:**
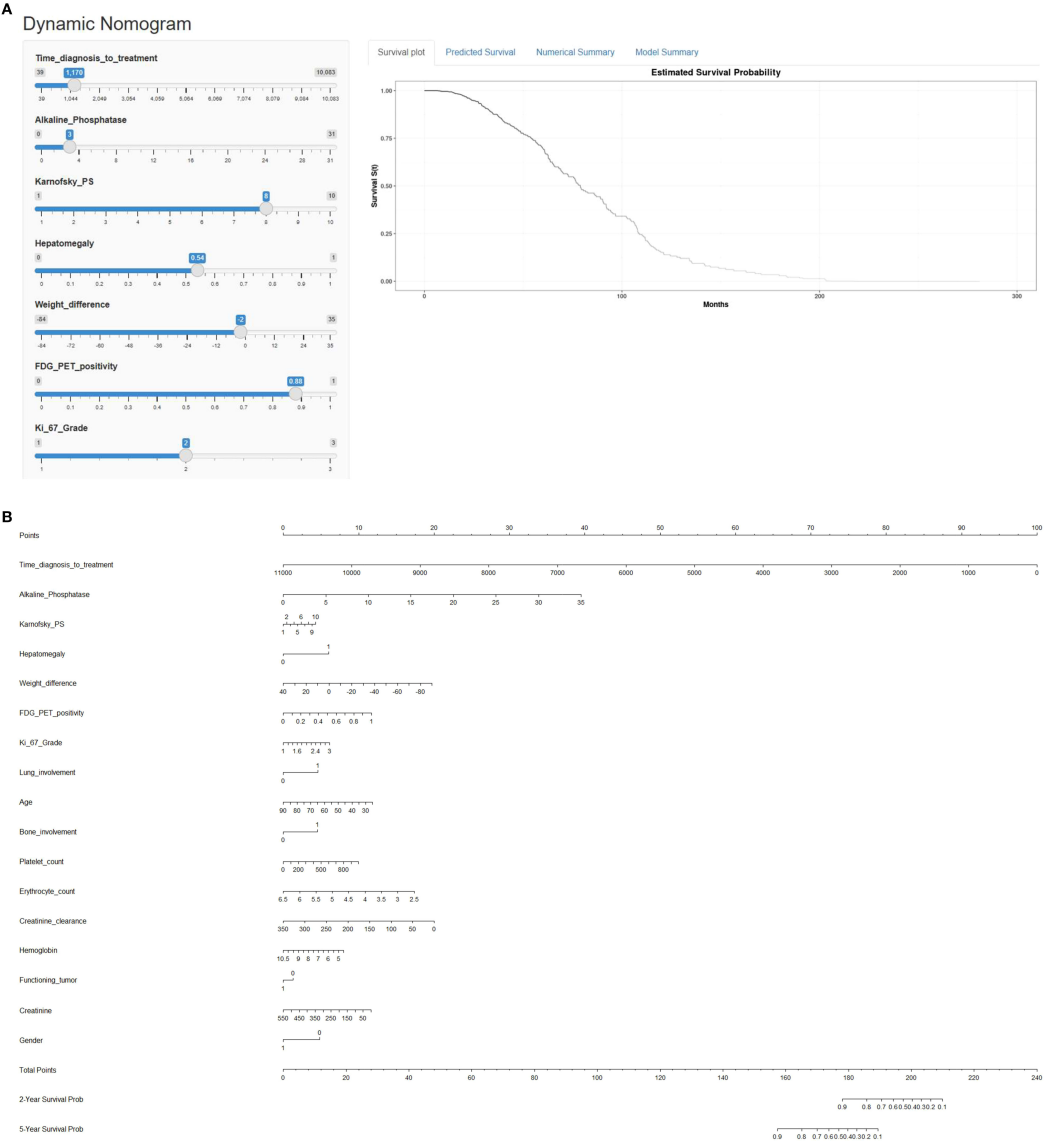
**(A)** Web-based survival rate calculator (Dynamic Nomogram (shinyapps.io)) to predict the overall survival of metastatic P-NEN patients treated with PRRT. Time_diagnosis_to_treatment refers to time from diagnosis to first PRRT treatment (in days). Alkaline phosphatase (ALP) values are shown in µkat/L, weight difference in kg, platelet count in G/L, erythrocyte count in T/L, creatinine clearance in mL/min/1.73 m² and creatinine in µmol/L. **(B)** Nomogram for prediction of overall survival (OS) in metastatic pancreatic NEN treated with PRRT. The nomogram is based on a cox proportional hazards model and is used by drawing a vertical line from each predictor value to the score scale at the ‘top-points’. After manually summing up the individual scores, the ‘total points’ correspond to the probability (prob) of overall survival, which are estimated by drawing a vertical line from this value to the bottom scale ‘2-year survival prob’ or ‘5-year survival prob’ to estimate overall survival.

**Figure 2A** depicts a web-based nomogram with these 17 selected relevant variables which is accessible through the URL: https://dynamicnomogramnet.shinyapps.io/dynnomapp/.

This is a simple-to-use web-based nomogram for convenient application, which can aid personalized treatment and clinical decision-making.

Values for the 17 prognostic variables can be chosen via horizontal sliders, which in turn computes individualized linear predictors from a Cox proportional‐hazards model, and dynamically renders (1) a Kaplan-Meier survival curve (2) numerical summaries, and (3) model parameter summaries in real time.

Another option is provided to predict overall survival at specific follow-up times, which subsequently can be viewed in the “Numerical Summary” tab.

**Figure 2B** depicts a predictive nomogram with these 17 variables constructed for manually calculating 2- and 5-year overall survival probabilities.

**Figure 3:**
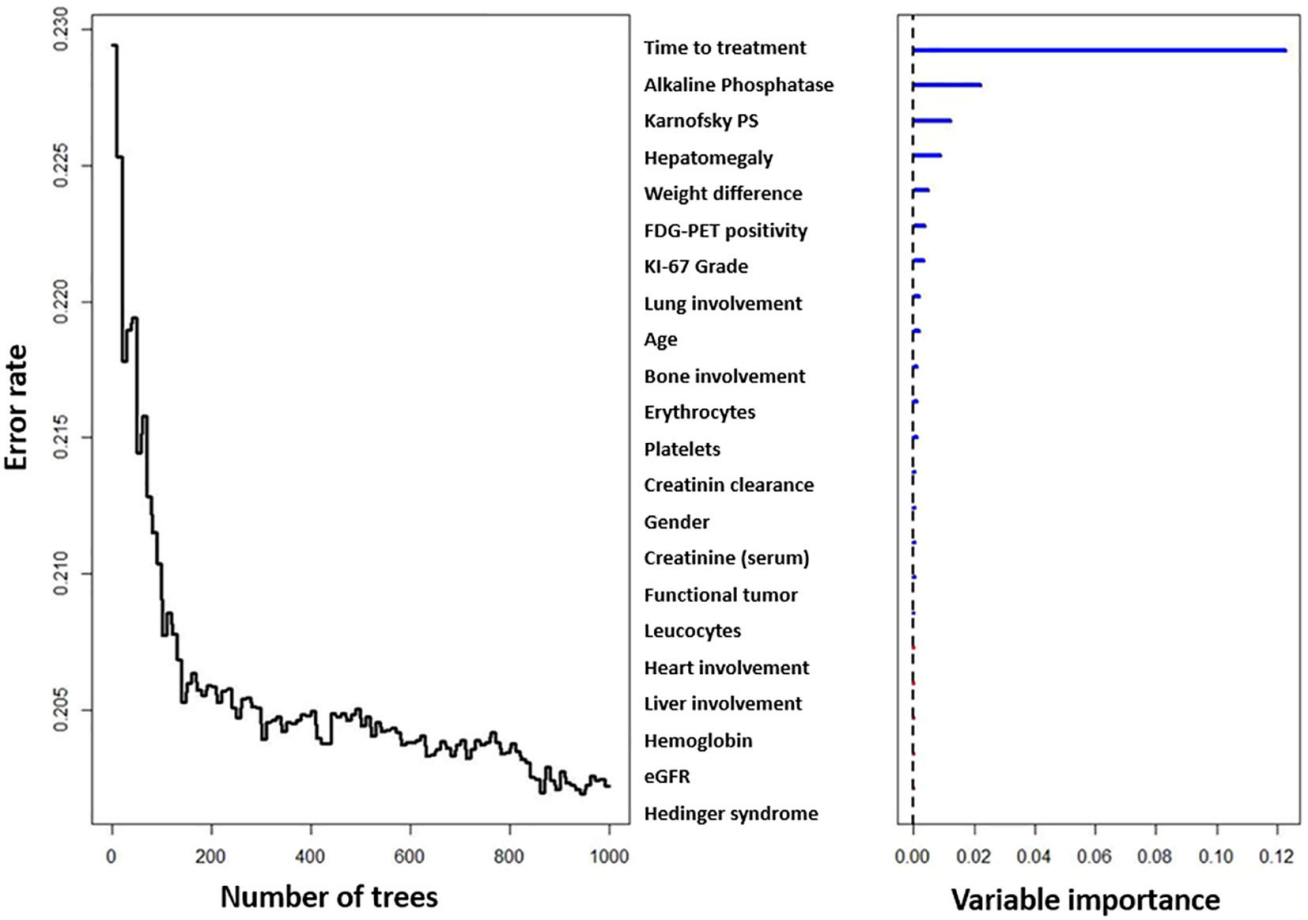
Variable importance and model error rate with increase in number of trees.

Time from diagnosis to first PRRT, alkaline phosphatase, KPS, presence of hepatomegaly, weight loss (unintentional loss of ≥2 kg weight in past 3 months), [_18_F]FDG-PET positivity (at variable timepoints), tumor grade based on proliferation index (Ki-67), presence of pulmonary metastases, age at PRRT, presence of bone metastases, platelet count, erythrocyte count, creatinine clearance, hemoglobin, functioning (functional) tumor, plasma creatinine, gender, presence of myocardial metastases, presence of liver metastases, presence of Hedinger syndrome (carcinoid heart disease), leucocyte count, and Modification of Diet in Renal Disease trial (MDRD)-based estimated glomerular filtration rate (eGFR) were according to their importance in the Random Survival Forest model all independent predictors for overall survival (**Figure 3**).

## Discussion

Pancreatic neuroendocrine tumors are a group of rare and heterogenous tumors with a poorly defined natural history and unclear biological behavior (25, 26).

Nowadays, P-NEN are being detected with increasing frequency and new treatment regimens including PRRT are being established. However, currently there is no set method to determine the prognosis of patients using the variable possible prognosticating parameters in the pre-PRRT setting.

In this study we have designed a single center, internally validated nomogram (PANEN-N) based on clinicopathological as well as imaging parameters for the prediction of overall survival (OS). In the RSF model, Time from diagnosis to first PRRT, alkaline phosphatase, KPS, hepatomegaly, weight loss, [_18_F]FDG-PET scan positivity, histologic grade, presence of lung metastases, age, presence of bone skeletal metastases, erythrocytes, platelets, creatinine clearance, hemoglobin, gender, functioning (functional) tumor, and creatinine were in order of importance all independent predictors for overall survival. This model had a high discriminative performance (AUC = 0.82) in the testing cohort.

Although several studies not specifically addressing PRRT or medical treatment have reported prognostic factors in the management of P-NENs (27), to our knowledge there is currently not a single nomogram in the literature identifying clinicopathological and imaging markers for clinical decision support in patients with metastatic P-NEN treated with PRRT in a meaningfully large cohort of patients. Furthermore, this study includes the largest number of pancreatic neuroendocrine neoplasm patients treated with PRRT at any single center and studied for prognostication of survival following PRRT with the aim of designing a predictive clinical support decision tool that could be used to include in the algorithm of informed consent by the patient.

The best way to sequence systemic therapeutic options in patients with P-NEN has not yet been fully established. For patients with unresectable disease, options to control tumor growth and symptoms related to tumor bulk or hormonal hypersecretion include somatostatin analogs, nonsurgical liver-directed therapy, and systemic antitumor therapy using everolimus or sunitinib, cytotoxic chemotherapy, or peptide receptor radionuclide therapy (PRRT).

A European phase III trial, SEQTOR (NCT02246127), is comparing the efficacy and safety of chemotherapy (fluorouracil and streptozotocin) followed by everolimus versus everolimus followed by fluorouracil and streptozotocin in patients with advanced and progressive pancreatic NET (28), and there is also an ongoing trial of PRRT versus sunitinib for progressive disease (29).

PRRT using radiolabeled somatostatin analogs is an option for patients with disease that expresses somatostatin receptors and has progressed on other treatment modalities including at least one somatostatin analog (30). Although clinical trials are being planned, there are no data yet specifically comparing PRRT with other therapeutic agents, and the choice of therapy in this situation has been previously based on the availability of PRRT and patient preference.

As evident from the nomogram, time to treatment is an important factor for these patients and reflects the apparent delay in the decision to perform PRRT since diagnosis. Time to treatment with PRRT could have been prolonged in several cases, since sequencing of earlier PRRT is not yet supported due to the lack of evidence based on phase-3, prospective, randomized clinical trials. Alkaline phosphatase (AP) is a known independent non-specific tumor marker and AP levels above normal have been reported as predictive of shorter survival in both univariate and multivariate analysis in patients with metastatic neuroendocrine tumors (31). Adriantsoa et al. reported on the prognostic value of AP in G1 and G2 NET patients, including 29 patients with duodenal/pancreatic NET, and reported that in multiparametric analysis progression-free survival correlated with serum AP level (p = 0.017) (32), thus emphasizing its significance as an independent prognostic marker. Furthermore, an elevated serum AP reflects the possibility of skeletal metastatic disease as well as the possibility of coexisting hepatic metastases. Skeletal metastases are not always easily appreciable on staging and restaging CT scans. Therefore, the value and trends in progression or regression of the alkaline phosphate should be monitored closely in comparison to SSTR-based PET/CT imaging and considered as a prognostic biomarker for patients with P-NEN.

KPS is a validated performance index of the physical ability of a patient and in a multivariate analysis Ezzidin et al. reported that KPS of less than or equal to 70 was an independent predictor of poor survival of GEP-NET patients treated with PRRT using [_177_Lu]Lu-DOTATATE (33). Weight loss of >2kg in past 3 months prior to the decision of commencement of PRRT was a marker of poor survival and this is also in line with previous studies (34) that reported baseline weight loss as a significant predictor of disease-specific survival in various GEP NET’s prior to radionuclide therapy with the radiolabeled somatostatin analog[_177_Lu]Lu-DOTATATE.

The presence of hypermetabolic tumor burden on an [_18_F]FDG PET/CT in histologically proven well-differentiated low-grade NEN represents tumor heterogeneity and is associated with either the pre-existence of aggressive tumor burden or dedifferentiation of disease during its course. In a prospective 10-year follow-up study in 166 patients with histologically proven gastroenteropancreatic neuroendocrine neoplasms (GEP-NEN) including 28 pancreatic NET patents ([_18_F]FDG negative n=7, a; [_18_F]FDG positive n=21), Binderup et al. demonstrated that a positive [_18_F]FDG PET scan was associated with a shorter OS than a negative [_18_F]FDG PET scan (hazard ratio: 3.8; 95% CI: 2.4– 5.9; P, 0.001). In G1 and G2 patients (n 5 140), a positive [_18_F]FDG PET scan was the only identifier of high risk for death (hazard ratio: 3.6; 95% CI, 2.2–5.9; P, 0.001). In this study, PRRT was performed in 78 (47% of enrolled) patients, and it was observed that in addition to a longer survival for the patients receiving PRRT, the survival benefit seemed most pronounced in the [_18_F]FDG –positive patients in whom the median survival time for those who received PRRT was 4.4 y compared with 1.4 y for patients not receiving PRRT (35). One of the first study to investigate the prognostic value of an integrated parameter derived from dual somatostatin receptor imaging and [_18_F]FDG PET in 24 (39%) pancreatic NET patients from a cohort of GEP-NET patients was performed by Chan et al, who developed the NETPET score, concluded that NETPET score was a significant predictor of overall survival on both univariate and multivariate analyses, emphasizing on the prognostic value of FDG positive tumor status of NET (12), findings which were later validated in a multicenter study (36).

Tumor proliferation index represented as Ki-67% defines the grade of tumor and is the basis for classification of neuroendocrine neoplasms. Several studies including patients with GEP-NEN receiving PRRT for G1 and G2 NET have not demonstrated any statistically significant prolongation in median overall survival with PRRT versus high-dose long-acting octreotide, a finding which has been reflected by the results of the NETTER-1 trial (37). This is most likely considered to be a result of the crossover of patients from standard-of-care arm to investigational therapy product arm on progression of disease over the prolonged follow up trial period. The well-differentiated lower grade (G1 and G2) of NEN (38) also represents the main group in which PRRT is usually recommended. In the largest cohort of intention to treat analysis of PRRT of NEN including n=1048 patients with WHO grades G1, G2, and G3 NEN, those with a lower Ki-67 index had a prolonged overall survival compared to higher grade neoplasms (15).

By the time the diagnosis of a NEN is established, most patients already have metastatic spread of disease, and out of all NEN, 40-50% of pancreatic NEN patients present with distant metastases at initial diagnosis (39). In general, NEN with distant metastases are considered incurable leading to a relatively shorter survival despite the currently available management options. In our study cohort, involvement of the lungs (pulmonary metastases) and bone metastases were found to be significant predictive factors for application toward the developed nomogram for OS.

In our study, younger age at diagnosis of pancreatic NEN was a predictor of improved overall survival status following PRRT. A recently published study explored the trends in the incidence and incidence-based mortality of early-onset GEP-NENs obtained from the Surveillance, Epidemiology, and End Results (SEER) database, and reported that the prognosis of early-onset GEP-NENs was significantly superior to that of later-onset GEP-NENs, regardless of the tumor site (40), where incidence-based mortality was analyzed for >1000 patients early-onset patients compared to >5400 later-onset patients (40). Although not directly related to post-PRRT survival assessment of pancreatic NEN patients, the results of the study provide an insight into the post-therapeutic survival trends in this patient population.

The current understanding in the development of PRRT related haematotoxicity, particularly myelodysplastic syndromes leading to poor survival prognosis is reported at <3% due to the relatively low estimated bone marrow absorbed radiation dose. In the analysis of long-term tolerability of PRRT in patients with neuroendocrine tumors, Bodei et al. (41) reported that risk factors associated with bone marrow toxicity were previous chemotherapy, other previous myelotoxic therapies and pre-existing anemia. When analyzing the codependent clinical variables, platelet toxicity grade was found to be a significantly associated factor with longer PRRT duration (41). In our study, of all hematological factors associated with overall survival post-PRRT, low platelet counts at diagnosis was of highest relevance followed by erythrocytopenia, and subsequently anemia. Here, it is important to note that hemoglobin and erythrocyte counts can be managed and sustained either with hemopoietic therapies such as erythropoietin and packed red blood cell transfusions, and low total and differential leukocyte counts can usually be managed with granulocyte colony stimulating factors. However, it is extremely challenging to manage significant or critical thrombocytopenia in clinical practice.

Renal irradiation arises from the proximal tubular reabsorption of the radiopeptide and the resulting retention in the interstitium. Due to their marked radiosensitivity to the range of doses resulting from PRRT, the kidneys represent the critical organs (42). Over prolonged time period, irrespective of nephroprotection, PRRT has the potential to affect the renal function with a median loss of creatinine clearance of up to 4% and 7% per year for 177Lu-octreotate and 90Y-octreotide, respectively. Risk factors promoting the decline of renal function after PRRT have been considered to be the cumulative/per-cycle renal absorbed dose, advanced age, hypertension, and diabetes (43). In our study, we observed that calculated plasma creatinine clearance value was comparatively more significant in the prognosis of overall survival post-PRRT compared to laboratory based estimated GFR.

Functional neuroendocrine neoplasms represent the group of patients that actively produce various hormones depending on the origin of the NEN and often associated with varying clinical symptoms depending on the metastatic spread of disease. It has been reported that functioning serotonin-producing P-NEN are aggressive neoplasms with a survival rate similar to that of other aggressive functioning neuroendocrine pancreatic neoplasms like ACTH-secreting P-NENs associated with Cushing’s syndrome (44).

Since most NETs are not functional (often not causing signs or symptoms), early diagnosis is difficult, and theoretically may reduce survival by reducing the chance of curative treatment. In this cohort with an individualized treatment regimen a slight survival benefit for non-functioning tumors (“1”) in comparison to functioning tumors (“0”) was found when all other variables were fixed. Indeed, our nomogram finds (when all other variables are fixed) a slight survival benefit for non-functioning tumors in comparison to non-functioning tumors.

Regarding patient gender, patient cohort treated with PRRT with pancreatic NEN in our study demonstrated a shorter overall survival for “1 (female)” compared to “0 (male), and this finding was inconsistent with the SEER database analysis, where compared to females, males had a better overall survival prognosis for tumors originating in the colon, small intestine, pancreas, and stomach (45).

Nomogram model reporting was done according to TRIPOD (Transparent Reporting of a multivariable prediction model for Individual Prognosis or Diagnosis)-standards (46). The TRIPOD offers a standard way for reporting the results of prediction modeling studies and thus aiding their critical appraisal, interpretation, and uptake by potential users. With the help of the TRIPOD model reporting, further correlative studies may be performed to cross-validate the results of this study with other centers, which would provide further statistical strength to these findings in different patient cohorts.

Our nomogram has, in our opinion, the potential to further aid clinical decision making for treatment with PRRT in patients with metastatic pancreatic NEN. It will provide P- NEN patients the opportunity to discuss their individual clinical situation based on the parameters analyzed and reported in the nomogram and empower them to make an informed decision for the management of their clinical condition with better understanding and greater confidence. For a wider clinical applicability and clinically practical benefit to patients and physicians, we plan to collaborate with other institutions, where a reasonable number of patients have been treated with PRRT, to collaborate with our dataset and cross-validate these findings.

Several limitations of this study were (I) The nomogram was not validated on an external dataset; (II) Our study includes patients from a single center, which is a limitation when considering the outcomes of this study. Perhaps, it would be interesting to look at PFS in future studies as it occurs earlier in the patient journey and may present the possibility of alternative therapeutic options, for example, it may indicate the use of localized therapies such as in case of liver or bone predominant progression, etc. However, focusing on the aim of our study, we would defer this to future sub-analyses. Finally (III), we did not identify differences in subgroups receiving different radiopharmaceuticals for PRRT (for e.g., [_177_Lu]Lu-DOTATOC vs[_90_Y]Y-DOTATATE).

Although progression free survival (PFS) is an often reported clinically relevant parameter, its relevance is usually limited to the early phase of survival analysis. However, the patients are always more interested in the more medically relevant survival outcome parameters, namely, overall survival (OS), which therefore has greater relevance for analysis and reporting. However, in this cohort, the OS plays a rather important role, as it is a more definitive parameter from patients’ perspective. Moreover, PFS in this group of patients may be misleading, since most patients were treated with PRRT as a last line therapy option, and PFS when measured via RECIST 1.1, which is primarily anatomical imaging based and unable to reliably measure bone disease, and therefore, has been debatable due to its obvious limitations in assessing targeted receptor based molecular imaging and therapy such as PRRT. Furthermore, there are currently no standardized, globally accepted protocols for response to assessment using PET/CT-based receptor-targeted molecular imaging. Moreover, despite certain variably sensitive tumor markers, there are no definitive, highly specific tumor markers for the evaluation of response to therapy or prognosis in patients with neuroendocrine neoplasms undergoing PRRT.

Prospects of this initial study include the validation of the current nomogram on one or several prospective cohorts, the addition of radiomics and deep learning imaging biomarkers to the current nomogram to better identify high survival P-NET groups with PRRT, and the generation of nomogram based on other clinically relevant outcomes such as PFS.

## Conclusion

This study proposes an internally validated nomogram to accurately predict overall survival for patients suffering from metastatic pancreatic neuroendocrine neoplasm based on the clinicopathological as well as medical imaging parameters, namely PANEN-N. The model could be used to facilitate decision support in daily clinical practice and can be used for patient counseling and shared decision making for patients presenting for peptide receptor radionuclide therapy as well as for generating new hypotheses. External multicenter validation of this nomogram is mandated prior to its routine clinical application.

## Data Availability

The original contributions presented in the study are included in the article/supplementary material, further inquiries can be directed to the corresponding author/s.
